
JOURNAL INFORMATION
==============================
NLM Title Abbreviation: ScientificWorldJournal
Journal ID: ScientificWorldJournal
NLM Journal ID: 101131163
Journal ID: TSWJ

The Scientific World Journal

ISSN: 2356-6140
EISSN: 1537-744X
Publisher: Wiley

ARTICLE INFORMATION
==============================
PMCID: PMC6009315
PMID: 29973849
DOI: 10.1100/tsw.2002.164
Article ID: 209537
Article version: 1
Subjects: Editorial

Founding Editorial: Cognition

Posner Michael
Publication date: 2002
Electronic publication date: 2002 Mar 15
Volume: 2
First page: 739
Last page: 739
Received 2002 Feb 12; Accepted 2002 Feb 13
Copyright: Copyright © 2002 Michael Posner.
Copyright year: 2002
License: This is an open access article distributed under the Creative Commons Attribution License, which permits unrestricted use, distribution, and reproduction in any medium, provided the original work is properly cited.
License URL: https://creativecommons.org/licenses/by/3.0/

Keywords: intelligence, emotions, social cognition, culture, language

==============================
Founding Editorial
*TheScientificWorldJOURNAL* (2002) 2, 739
ISSN 1537-744X; DOI 10.1100/tsw.2002.164

### Founding Editorial: Cognition

Michael Posner
*Principal Editor, Cognition*

*Received February 12, 2002; Accepted February 13, 2002; Published March 15, 2002*

**KEY WORDS:** intelligence, emotions, social cognition, culture, language

**DOMAINS:** cognition

The field of Cognition involves the scientific study of mental functions including their emotional, social, and cultural context. Under this definition, many academic disciplines such as Anthropology, Computer Science, Linguistics, Philosophy, and Psychology are appropriate. We will coordinate our work closely with the Higher Level Brain Function Domain when dealing with papers with a cognitive neuroscience focus.

*TheScientificWorldJOURNAL* should be a vehicle for publishing promptly, carefully reviewed papers in this vast and in some areas swiftly moving field. In submitting our first paper to *TheScientificWorldJOURNAL* (Attentional Phenotypes for the Analysis of Higher Mental Function, *TheScientificWorldJOURNAL* (2002) **2**, 217–223) I found the two months from initial submission to publication very refreshing and the two thoughtful reviews among the most helpful I have received. We hope to provide the same service to others. While not all papers can be published, we would like to be prompt, clear, and helpful in dealing with each submission.

As an electronic Journal, we hope to attract submissions from all parts of the world and help serve to develop a worldwide community of researchers dedicated to the study of cognition.

**This article should be referenced as follows:**

Posner, M. (2002) Founding editorial: Cognition. *TheScientificWorldJOURNAL* **2**, 739.

Email: mip2003@med.cornell.edu
©2002 with author.
